# Clinical Relevance of Distinguishing Between Three Endoscopy-Based Conditions, Bronchiectasis, Bronchomalacia, and Their Combination in Dogs: A Retrospective Study

**DOI:** 10.3390/vetsci12050487

**Published:** 2025-05-18

**Authors:** Aurélie Lyssens, Géraldine Bolen, Aline Fastrès, Cécile Clercx, Frédéric Billen

**Affiliations:** 1 Department of Clinical Sciences, Faculty of Veterinary Medicine, B67 Sart Tilman, University of Liege, 4000 Liege, Belgium; gbolen@uliege.be (G.B.); cclercx@uliege.be (C.C.); fbillen@uliege.be (F.B.); 2 Centre Hospitalier Vétérinaire Anicura Nordvet, 1 Rue Delesalle, 59110 La Madeleine, France; aline.fastres@anicura.fr

**Keywords:** bronchiectasis, bronchomalacia, endoscopy, dogs

## Abstract

Bronchiectasis (BE) and bronchomalacia (BM) are chronic respiratory diseases in dogs, yet their combined occurrence (BEBM) is not well studied. This retrospective study analyzed 65 dogs diagnosed via endoscopy with BE, BM, or BEBM (E-BE, E-BM, E-BEBM) to identify clinical and pathological differences and assess how imaging results (radiography and computed tomography (CT)) align with endoscopic findings. Clinical symptoms like coughing, dyspnea, and exercise intolerance were similar across all groups, except lung crackles, which were more common in E-BEBM. Inflammation seen during bronchoscopy and bronchoalveolar lavage fluid results, including neutrophil counts, showed no significant differences between groups. Bacterial infections were present in 15% of dogs with no difference among groups. Diagnostic agreement between radiography and endoscopy was low: 18.1% for E-BE, 10.5% for E-BM, and 38.4% for E-BEBM. CT results matched endoscopic findings in all E-BE cases but only in half of E-BM and 40% of E-BEBM cases. The bronchial-to-arterial ratio, a benchmark for BE diagnosis, did not align with CT findings. Overall, the study found limited clinical or pathological differences between BE, BM, and BEBM and limited concordance between imaging and endoscopic findings, emphasizing the need for further research to clarify potential implications for treatment strategies.

## Simple Summary

The co-occurrence of bronchiectasis and bronchomalacia (BEBM) in dogs remains underexplored. In 65 dogs diagnosed with bronchiectasis (BE), bronchomalacia (BM), or BEBM, we aimed to identify potential clinicopathological differences among these conditions and to assess the concordance between imaging modalities. Our findings revealed minimal clinicopathological differences between endoscopy-based diagnoses of BE, BM, and BEBM, as well as a lack of significant differences in bronchoalveolar fluid analysis across the three groups. Diagnostic agreement between imaging modalities and bronchoscopy was limited. These observations call into question the clinical relevance of distinguishing among these diagnoses.

## 1. Introduction

Bronchiectasis (BE) is a common chronic lung disease in people and dogs, which is characterized by a progressive and irreversible dilatation of the bronchial wall and associated with chronic inflammation and infection [1,2,3,4]. Similar to human medicine, the pathogenesis of BE in dogs is unclear [5]. It is believed that BE involves a complex interplay of chronic infection, inflammation, and impaired mucociliary clearance [3,6]. Dynamic bronchomalacia (BM) is also a common airway disease in dogs and has recently been redefined as a regional to diffuse dynamic airway collapse of segmental bronchi, subsegmental bronchi, or both [7]. As for BE, the exact underlying mechanism of canine BM is still unknown, but infection and inflammation could possibly play a role, as well as degenerative changes [2,8]. In human medicine, a combination of BE and BM (BEBM) has been reported sporadically [9,10] in which airway inflammation in BE is believed to potentiate airway collapse due to bronchial wall weakness [10]. In dogs, concurrent airway collapse has been described in dogs with BE [5], and the authors’ experience indicates that a combination of BE and BM is often observed in the same patient during bronchoscopy. However, a combined BEBM entity has not been well documented.

In dogs, a diagnosis of BE and BM involves thoracic imaging and bronchoscopy [1,7]. For diagnosing BE, computed tomography (CT) and bronchoscopy are generally considered as the preferred diagnostic methods [1,11]. Using CT, measurement of the bronchial-to-arterial ratio (BAR) is the most widely used criterion for detecting bronchial dilatation, with BAR values of ≥2.0 during the expiratory phase suggesting evidence of bronchiectasis [11]. Bronchoscopic diagnosis of BE is based on the observation of one or more dilated bronchi [5,12]. For diagnosing BM, bronchoscopy is considered the gold standard, showing flattening and collapse of the airway lumen [7,8,13]. Other common bronchoscopy findings in dogs with BM include airway inflammation and mucus accumulation [8]. CT is also useful to diagnose BM, but both the inspiratory and expiratory phases need to be explored to highlight dynamic collapse [7]. Since the pathogenesis of BE and the inflammatory, infective, and molecular drivers of disease progression are not yet fully understood in both human and veterinary medicine, development of an ideal treatment protocol or of novel treatment options is challenging [5,14]. Current human treatment strategies for BE focus on airway clearance and anti-inflammatory medications, including oral or inhaled corticosteroids, and antibiotics [15]. However, no guidelines exist for the ideal treatment protocol for neither dogs with BE nor for dogs with BM. Despite conflicting evidence regarding the presence and role of bacterial infection in the pathogenesis of both BE and BM, antibiotics are frequently administered empirically to dogs with these conditions [1,5,7]. This contradicts the One Health approach and the need for reduced antibiotic use. To better guide clinicians in treating BE and BM, more knowledge and insight into the pathogenesis, the role of bacterial and inflammation in both conditions are needed.

Dogs with BE, dynamic generalized BM, and BEBM, based on bronchoscopic criteria, were retrospectively included in this study. The primary objective was to describe the clinicopathological characteristics across the three groups to assess whether these conditions should be considered distinct entities, warranting different management strategies in the future. A secondary aim was to compare the imaging findings of the affected dogs with the corresponding bronchoscopic results.

## 2. Materials and Methods

### 2.1. Case Selection and Data Collection

Medical records from client-owned dogs, presented to the Small Animal Teaching Hospital, Liège, Belgium, between November 2014 and November 2021 that underwent bronchoscopy with a bronchoalveolar lavage and for which videos during bronchoscopy were obtained were retrospectively reviewed. Data collected included age, sex, breed, body weight, duration, and severity (mild, moderate, or severe) of clinical signs (chronic cough, exercise intolerance, dyspnoea), abnormalities during physical examination (increased lung sound and crackles on lung auscultation), any previous treatment, ongoing treatment, results of the blood examination (complete blood count, biochemistry, electrolytes, and C-reactive protein (CRP)), results of Baermann analysis, complete description of bronchoscopy and bronchoalveolar lavage fluid analysis (including bacterial culture and quantitative polymerase chain reaction (qPCR) analysis), results of thoracic radiography (RX), CT, and echocardiographic findings.

### 2.2. Bronchoscopy

**Figure 1:**
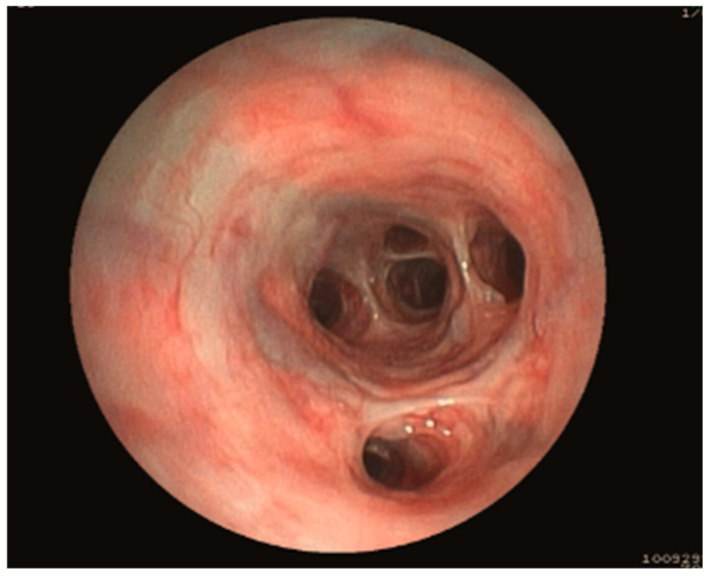
Example of bronchiectasis during bronchoscopy. An obvious macroscopic lack of tapering or increased diameter of the bronchial lumen is observed.

All videos made during bronchoscopy were double-blind reviewed by 2 independent diplomates of the European College of Veterinary Internal Medicine. Only dogs with video recordings of sufficient quality were considered. Each dog was classified into 3 different groups based on endoscopy: E-BE in the presence of bronchiectasis only, E-BM in the presence of bronchomalacia only, and E-BEBM in the presence of both bronchiectasis and bronchomalacia. E-BE was defined as an obvious macroscopic lack of tapering or increased diameter of the bronchial lumen when compared to adjacent bronchi (Figure 1).

**Figure 2:**
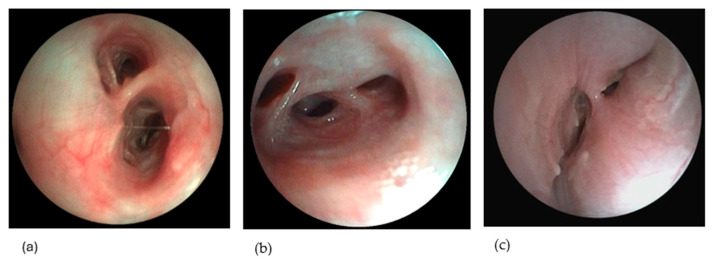
Examples of bronchomalacia during bronchoscopy. Collapse of the bronchi is observed. Based on the endoscopic grading system of [7], a subclassification of bronchomalacia was made. (**a**) Subclassification (BE)BM-I, whereby a 25–50% reduction in airway diameter is observed. (**b**) Subclassification (BE)BM-II, whereby a 50–75% reduction in airway diameter is observed. (**c**) Subclassification (BE)BM-III, whereby a > 75% reduction in airway diameter is observed.

E-BM was defined as dynamic airway collapse of the bronchi whereby changes in luminal diameter were observed during respiration (Figure 2a–c). E-BE and E-BM were considered diffuse if observed in minimum two lobes. Dogs with localized E-BE or E-BM (i.e., in only 1 lobe), dogs with E-BM with less than 25% reduction in bronchial lumen diameter, dogs with static E-BM (defined as persistent reduction in luminal diameter during all phases of respiration) such as dogs with brachycephalic obstructive airway syndrome, and lastly, dogs diagnosed with a grade III or IV tracheal collapse were all excluded from the study. Dogs diagnosed with E-BM were subclassified into 3 subcategories based on a previously described grading system [7]: E-(BE)BM-I (25–50% reduction in airway diameter; Figure 2a), E-(BE)BM-II (50–75% reduction in airway diameter; Figure 2b) and E-(BE)BM-III (>75% reduction in airway diameter; Figure 2c).

**Figure 3:**
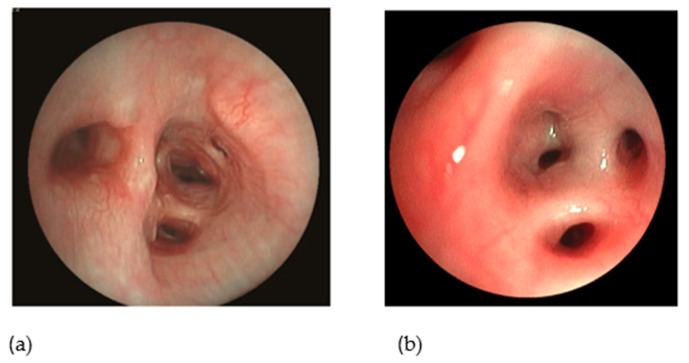
Evaluation of bronchial inflammation based on bronchoscopy. (**a**) Mild to moderate bronchial inflammation was defined as the presence of erythema of the bronchi. (**b**) Severe bronchial inflammation was defined as the presence of a thickened, oedematous, and erythematous bronchial mucosa.

The presence and severity of bronchial inflammation (mild to moderate versus severe) was noted. Mild to moderate bronchial inflammation was defined as the presence of erythema of the bronchial mucosa (Figure 3a). Severe bronchial inflammation was defined as the presence of a thickened, oedematous, and erythematous bronchial mucosa (Figure 3b).

### 2.3. Bronchoalveolar Lavage Fluid Analysis

Bronchoalveolar fluid (BALF) analysis results were retrospectively reviewed and included cytology, total cell count (TCC), differentiated cell count (DCC), bacterial culture, and qPCR results. Total cell count was calculated using a haemocytometer at 2 occasions and averaged. Normal TCC was considered equal to or less than 600 cells per microliter [16]. Cytospin preparations, prepared by cytocentrifugation (centrifugation at 197× *g* for 4 min at 20 °C, Thermo Scientific Cytospin 4 (Waltham, MA, USA), were stained with Diff Quick^®^ to assess cytology and DCC. DCC was performed by counting 100–300 nucleated cells with an oil-immersion objective lens at 100× magnification. Slides were examined for presence of signs of inflammation and intra- and extracellular bacteria. Neutrophilic inflammation was concluded if >12% of neutrophils were observed [16]. TCC and DCC were established by 1 experienced veterinarian and 1 independent lab technician. DCC and cytospin slides were not re-evaluated in this retrospective study. Semi-quantitative aerobic culture testing was performed by a commercial veterinary diagnostic laboratory (Antech, Liège, Belgium). Finally, qPCR testing for *Bordetella bronchiseptica*, *Mycoplasma cynos*, *Angiostrongylus vasorum*, and *Crenosoma vulpis* was performed at the clinical pathology laboratory (Department of Veterinary Pathology, Liège, Belgium). Infection was defined as an increased TCC, neutrophilic inflammation, and presence of intra- or extracellular bacteria and/or positive culture (>1.7 × 10^3^ colony-forming units (CFU) per milliliter of BALF) and/or positive qPCR results (cycle threshold < 34) [17].

### 2.4. Diagnostic Imaging

**Figure 4:**
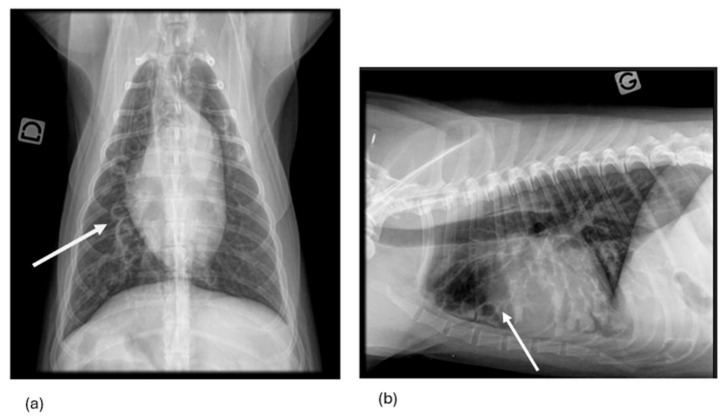
Ventrodorsal (**a**) and left-lateral (**b**) radiographs of a dog with bronchiectasis. Bilaterally (right > left), a severe saccular bronchiectasis can be observed (white arrow), as well as a diffuse bronchial pattern. The peripheral parts of the bronchi are dilated with a liquid content. Other abnormalities on these radiographs include an alveolar pattern cranioventrally in the right middle lung lobe, mild tracheal flaccidity, and presence of air in the esophagus. D means “right”.

All RX and thoracic CT were systematically reviewed by a board-certified radiologist unaware of the dog’s group. Right lateral and ventrodorsal RX, not standardized for the respiratory phase, were reviewed for indications of BE and BM. BE was diagnosed based on radiography (R-BE) in presence of non-tapering airways and further defined as saccular or cylindrical BE (Figure 4) [4]. Saccular BE referred to dilatation of the bronchi with focal saccular dilatations [4]. Cylindrical BE referred to dilatation of the bronchi without tapering toward the periphery [4].

**Figure 5:**
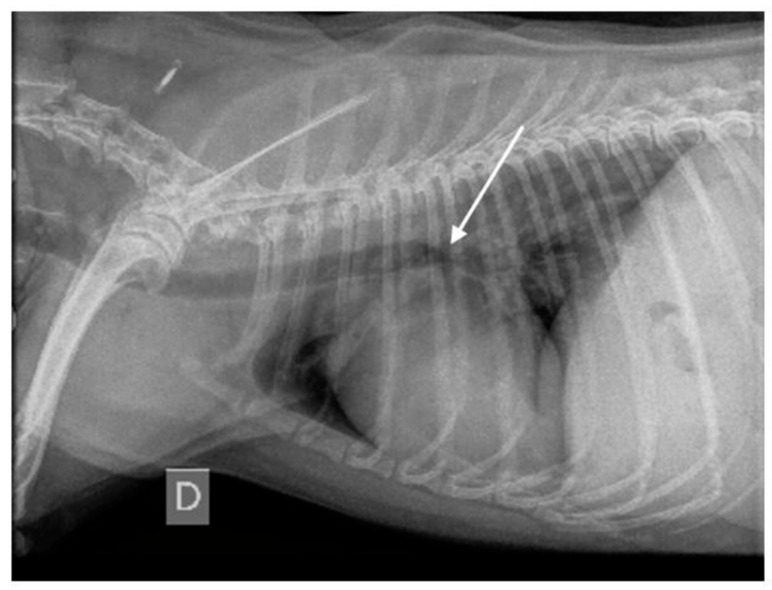
Right-lateral thoracic radiograph of a dog with bronchomalacia. A collapse of the carina can be observed (white arrow). D means “right”.

Since spare information exists regarding radiographic criteria for diagnosing BM, R-BM was diagnosed on RX when narrowing of the carina was present [18] (Figure 5).

Collapse of the carina was identified as either absent or present. Other recorded radiographic findings included pulmonary consolidation, bronchial wall thickening, and bronchial occlusion.

Thoracic CTs, using a two-phased breath-hold technique (during inspiration with airway pressure maintained at 15 to 20 cm of water and during a respiratory pause), were evaluated for the presence of BE and BM (CT-BE and CT-BM). Dogs were sedated with butorphanol, induced with propofol, and anaesthesia was maintained with isoflurane gas and 100% oxygen. For acquisition of the thoracic CT, the dogs were placed in sternal recumbency, under general anaesthesia after a transient period of hyperventilation to induce apnoea and to avoid motion artifacts. Two different scanners were used: a 16- and a 64-multislice scanner before and after April 2019, respectively. Acquisition parameters used were as follows for the 16-multislice CT scanner: tube voltage 120 kV, reference tube current 130 mA, and pitch 0.7–1.15. Scan tube current was modulated by automatic exposure control (Care Dose, Siemens Medical Solutions, International). Image data sets were reconstructed using parameters of 200–300 mm field of view, 512 × 512 matrix, 1 mm slice thickness, and B60f reconstruction algorithm (window level −500 and window width of 1500) with filter back projection. Acquisition parameters for the 64-multislice CT scanner were as follows: tube voltage 120 kV, reference tube current 170 mA, and pitch 0.8 to 1.2. Scan tube current was modulated by automatic exposure control (Care Dose, Siemens Medical Solutions, International). Image data sets were reconstructed using parameters of 300 mm field of view, 512 × 512 matrix, 1 mm slice thickness, and Br59 reconstruction algorithm (window level −500 and window width of 1500) with iterative reconstruction.

**Figure 6:**
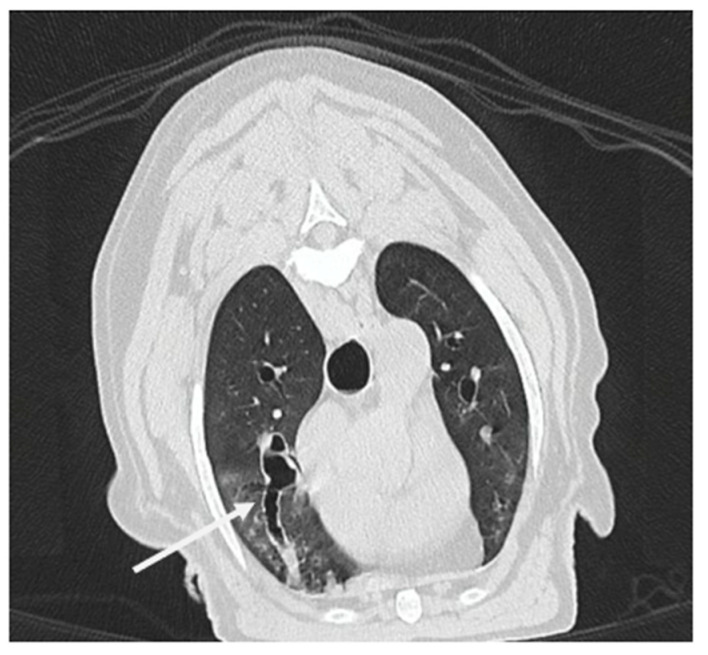
Transverse computed tomography image (lung window) in expiratory phase of a dog with severe bronchiectasis. Presence of an irregular dilation in longitudinal section of the stem bronchus of the right cranial lung lobe (white arrow). This dilation is associated with bronchial wall thickening along the entire length and takes on a saccular appearance toward the periphery. A heterogeneous increase in lung parenchymal attenuation is also present, taking on a ground-glass opacification.

CT-BE was defined by the presence of non-tapering airways with either saccular or cylindrical BE (Figure 6) [19].

**Figure 7:**
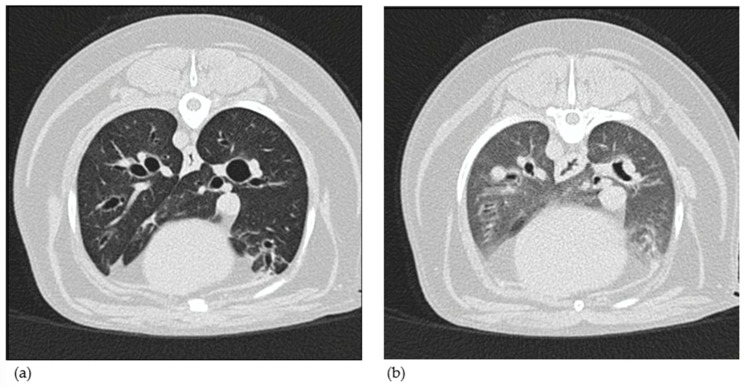
Computed tomography in inspiratory phase (**a**) and expiratory phase (**b**) of a dog with severe bronchomalacia (>75% on bronchoscopy). A dynamic bronchial collapse can be noted, especially at caudal stem bronchi (clear reduction in diameter and flattening during expiration). The other bronchi also decrease in diameter during expiration, but less markedly. A heterogeneous increase in lung parenchymal attenuation is also present, taking on a ground-glass opacification.

Additionally, but not for diagnostic purpose, BAR was measured as described in the article of [11] for dogs with an endoscopic diagnosis of BE and BEBM. Measurements of the BAR for the right and the left cranial lobar bronchi were evaluated at the level of the 4th rib on cross-section images and compared to its corresponding artery. The BARs for the right middle lung lobe and the caudal part of the left cranial lung lobe were measured at the level where the bronchus was at its largest diameter, on long-axis orientation, and compared to its corresponding artery. The BAR for the right accessory lung lobe was measured at the level of the bifurcation of the right caudal lobar bronchus and the accessory lobar bronchus and compared to its corresponding artery. The bronchial diameter was measured at the inner part of the bronchus during inspiration and expiration. The BAR for the right and left caudal lobar bronchi was not assessed in this study. CT-BM was defined by the presence of airway collapse of the main bronchi and segmental bronchi [20] (Figure 7).

Other recorded CT findings included pulmonary consolidation, bronchial wall thickening, bronchial occlusion, and ground-glass opacification.

### 2.5. Final Aetiology

Based on all available data, a final diagnosis was attributed to each dog. Dogs were diagnosed with eosinophilic bronchopneumopathy if they exhibited a moderate to severe bronchointerstitial pattern, abundant yellow-green mucopurulent material on bronchoscopy, and more than 50% eosinophils in the BALF [21]. A diagnosis of parasitic bronchitis was made when parasites were observed either during bronchoscopy, identified in BALF analysis by qPCR, or detected through a Baermann test.

Dogs were suspected to have idiopathic pulmonary fibrosis based on the signalment, clinical signs at presentation, compatible CT findings, exclusion of other cardiopulmonary diseases, and histopathology of the lungs, if available [22]. Lastly, dogs were diagnosed with chronic bronchitis if they were coughing for more than 2 months and for which no underlying specific cause could be identified [23].

### 2.6. Statistical Analysis

Statistical analyses were conducted using SAS (version 9.4) software. Categorical variables were summarized using frequency tables, presenting both the number and percentage of occurrences. Continuous variables were described using the mean and standard deviation (±SD), the median and first and third quartiles [Q1; Q3], and the range of extreme values (Min; Max). The 3 groups (BE, BM, and BEBM) were described and compared for the signalment of the dogs and their clinical signs (cough severity, presence of dyspnoea, presence of crackles on lung auscultation), and the remaining study variables were performed. A Chi-square test was used for categorical variables, while a non-parametric Kruskal–Wallis test followed by Dunn’s post hoc tests with Bonferroni correction were applied for continuous and ordinal variables. In order to examine the relationship between the continuous variables (neutrophil percentage or TCC) and a categorical variable (degree of inflammation for example), a non-parametric Kruskal–Wallis test was applied. The relationship between qualitative variables was analyzed using the Chi-square test to measure the association between them (presence of positive bacterial culture and the degree of inflammation). A paired Student’s *t*-test was used to assess significant differences in BAR measurements between the inspiratory and expiratory phases. If no statistical differences were noted between the three groups regarding their clinicopathological findings and BAR measurements, no additional details were provided about the subclassification within each BM group (BM-I, BM-II, BM-III, BEBM-I, BEBM-II, and BEBM-III). The results were considered significant at the 5% threshold (*p* < 0.05).

## 3. Results

### 3.1. Description of the Clinicopathological Findings

#### 3.1.1. Group Allocation Based on Bronchoscopy

Based on the medical reviews, 97 dogs were initially included. Twenty dogs were excluded, since only images of bronchoscopy were available instead of videos. Nine dogs were excluded since they had a reduction in airway diameter of <25%. Three dogs were excluded after being diagnosed with a grade III tracheal collapse. After double-blinded review of the bronchoscopy videos, a total of 65 dogs met the inclusion criteria. In 16 of those 65 dogs, there was an interobserver disagreement. In 5/16 dogs, this interobserver disagreement was linked to the classification in either E-BM-I or E-BM-II in 2 dogs and in either E-BM-II or E-BM-III in 3 dogs. In the remaining 11 cases, the disagreement was related to the presence or absence of BE. All the bronchoscopy videos were reviewed once more by both observers together, and a consensual appraisal was performed blindly from previous assessments. Of the 65 dogs, 16 dogs were classified in the E-BE group, 31 in the E-BM group (10, 11, and 10 in BM-I, BM-II, and BM-III, respectively), and 18 in the E-BEBM group (5, 11, and 2 in BEBM-I, BEBM-II, and BEBM-III, respectively) (Table 1).

#### 3.1.2. Study Population

The mean age of dogs for all groups was 9.6 years ± 3.1. No significant statistical difference in the mean age among the three groups (8.7 years ± 3.5 in E-BE, 9.9 years ± 3.1 in E-BM, and 10 years ± 2.4 in E-BEBM) was present (*p* = 0.44). There were 31 female dogs (2 intact and 29 neutered) and 34 male dogs (14 intact and 20 neutered). Overall, a total of 24 breeds was represented in this study (Table 1). Two dogs (dog 21 and dog 23) received an antibiotic treatment within 2 weeks prior to presentation, and only one dog (dog 21) was still receiving treatment at the time of presentation. Thirteen dogs (dog 6, 14, 32, 34, 35, 39, 40, 41, 44, 45, 53, 59, and 65) received an anti-inflammatory dosage of steroidal anti-inflammatory drugs (prednisolone or dexamethasone) within 2 weeks prior to presentation and all were still on this medication at the time of evaluation.

#### 3.1.3. Clinical Signs

Clinical signs for each group are reported in Table 2. The duration of the clinical signs varied from 1 month to 48 months (mean 10.3 months). Cough, severe (50.8%) or moderate (49.2%), was observed in all dogs. Exercise intolerance was also observed in 47.7% of dogs, while expiratory dyspnea was a clinical complaint in 30.8% of the cases. On pulmonary auscultation, increased lung sounds were present in 13.8% of dogs, and lung crackles were auscultated in 27.7% of dogs. The only significant difference between the three groups was the presence of lung crackles (*p* = 0.017), which was more frequently auscultated in E-BEBM (50%) compared to E-BM (25.8%) and E-BE (6.3%). No significant differences were found regarding the severity of cough (*p* = 0.57), presence of dyspnea (*p* = 0.13), exercise intolerance (*p* = 0.091), or increased lung sounds (*p* = 0.097) between the three groups.

#### 3.1.4. Blood and Fecal Analysis

Complete blood count was normal in 90.7% of dogs. Mild leukocytosis with neutrophilia was present in seven cases, and mild eosinophilia was present in two cases. Serum biochemistry revealed no abnormalities in 93.8% of cases. In the remaining dogs, alkaline phosphatase values were increased from two up to five times the upper reference limit. All these dogs had a history of recent prednisolone treatment. CRP was measured in only four dogs, with increased CRP values in three dogs (dog 21, dog 48, and dog 57) ranging from 15 mg/L to 26.4 mg/L (upper reference limit: 9 mg/L). The Baermann test was performed in 15.4% of dogs and was positive once for *Crenosoma vulpis* in dog 1.

#### 3.1.5. Bronchoscopy Findings

Mild to moderate macroscopic bronchial inflammation was observed in 34 dogs, including 19 dogs with E-BM, 9 dogs with E-BE, and 6 dogs with E-BEBM. Severe inflammation was noted in 11 dogs, including 2 dogs with E-BE, 5 dogs in the E-BEBM group, and 4 with E-BM. However, no significant difference was noted in the degree of macroscopic inflammatory aspect of the bronchial mucosa (mild/moderate and severe) between the 3 groups (*p* = 0.18). Even after excluding dogs that had received prednisolone prior to presentation, the severity of the macroscopic inflammatory aspect of the bronchial mucosa on bronchoscopy remained comparable across the three groups (*p* = 0.27).

#### 3.1.6. BALF Cytological and Microbiological Analysis

The median TCC was 1100.0 cells/µL [720.0; 2000.0] for all three groups and 1012.0 cells/µL [752.5–1560.0], 1160 cells/µL [720.0–2015.0], and 1220.0 cells/µL [720.0–2150.0] for E-BE, E-BM, and E-BEBM, respectively. No significant difference was noted in the median TCC between any group (*p* = 0.89). The median percentage of neutrophils was 20% [10.0–50.0] for all three groups and 20% [10.0–65.0], 15% [7.0–30.0], and 39.5% [13.0–75.0] for E-BE, E-BM, and E-BEBM, respectively, but those percentages did not differ significantly between groups (p = 0.087). Even after excluding dogs receiving antibiotics at presentation and/or receiving prednisolone upon presentation, no significant differences in the median neutrophil percentage in BALF were observed between the three groups (*p* = 0.088 and *p* = 0.084, respectively). The median percentage of neutrophils varied significantly according to the degree of macroscopic inflammation of the bronchial mucosa (*p* = 0.0053). In contrast, no significant association was found between the TCC and the degree of inflammation (*p* = 0.72). An increase in percentage of eosinophils (>10%) was only seen in dogs diagnosed with eosinophilic bronchopneumopathy and in the dog with parasitic bronchitis.

Ten dogs were diagnosed with a bacterial infection based on culture (eight dogs) and qPCR (two dogs). In all eight cases, culture demonstrated the presence of a single bacterium. Organisms identified in each group are summarized in Table 3. There was no significant difference in the presence of concomitant bacterial infection among the three groups, whether including dogs receiving antimicrobials at presentation (*p* = 0.081) or excluding them (*p* = 0.10). Additionally, no significant association was observed between the presence of bacterial infection in BALF and the degree of inflammation observed during endoscopy (*p* = 0.29), even after excluding dogs receiving antibiotics at presentation (*p* = 0.32).

#### 3.1.7. Thoracic Radiography Findings

RXs were available for review in 43/65 dogs. In the remaining 22 dogs, RXs were taken by the referring veterinarian and not available for review. A summary of the RX findings is available in Table 1. Based on the RXs, a diagnosis of R-BE, R-BM, and R-BEBM was made in 11, 6, and 16 dogs, respectively. In 10 dogs, a diagnosis of neither R-BE, R-BM, nor R-BEBM was made based on the available RXs. Pulmonary consolidation was observed in three dogs with E-BE, six with E-BM, and six with E-BEBM. Bronchial wall thickening was noted in 10 dogs with E-BE, 17 with E-BM, and 11 with E-BEBM. Bronchial occlusion was observed in two dogs with E-BE, two with E-BM, and three with E-BEBM.

#### 3.1.8. Computed Tomography Findings

A thoracic CT with inspiratory and expiratory breath holds was performed in 13/65 dogs. The other dogs had either no CT performed (46 dogs), a CT performed only under sedation (1 dog), or with only one respiratory phase available (5 dogs). A summary of the CT findings is available in Table 1. Based on the CTs, a diagnosis of CT-BE, CT-BM, and CT-BEBM was made in two, seven, and four dogs, respectively. Pulmonary consolidation was observed in one dog with E-BE, two with E-BM, and three with E-BEBM. Bronchial wall thickening was noted in two dogs with E-BE, five with E-BM, and five with E-BEBM. Bronchial occlusion was observed in two dogs with E-BE, one with E-BM, and no dog with E-BEBM.

Sixty BAR measurements were calculated from various locations (i.e., five lobes) during both the inspiratory and expiratory phases from all six dogs with a CT diagnosis of BE and BEBM (Table 4). The difference in mean BAR measurements between the inspiratory and expiratory phases was evaluated in dogs with CT-BE and CT-BEBM. In dogs with CT-BE and CT-BEBM, no statistically significant differences were observed in the mean BAR measurements across both respiratory phases for all five lobes. However, when considering all dogs together (CT-BE and CT-BEBM), significant differences in the mean BAR measurements between the inspiratory and expiratory phases were observed in the left cranial lung lobe (*p* = 0.015), right cranial lung lobe (*p* = 0.032), and right middle lung lobe (*p* = 0.047).

#### 3.1.9. Final Etiology

Overall, chronic bronchitis was the most common final diagnosis (55/65 dogs) in all three groups. Eosinophilic bronchopneumopathy (n = 5), idiopathic pulmonary fibrosis (n = 4), and parasitic bronchitis (n = 1) constituted the other final diagnoses (Table 1). Histopathology was performed in only one dog and confirmed idiopathic pulmonary fibrosis.

### 3.2. Comparison of the Imaging-Based Diagnosis with the Endoscopy-Based Diagnosis

#### 3.2.1. Thoracic Radiography Versus Endoscopy

Among the 11 dogs with E-BE, only 2 were also diagnosed with R-BE (18.1%). Similarly, of the 19 dogs with E-BM, 2 had a corresponding diagnosis of R-BM (10.5%). Finally, among the 13 dogs with E-BEBM, 5 were identified as R-BEBM (38.4%). No significant associations were observed for any of the RX findings (non-tapering airways and carina collapse) and the endoscopic diagnoses (*p* = 0.81 and *p* = 0.73, respectively).

#### 3.2.2. Thoracic CT Versus Endoscopy

Thoracic CTs were available in two dogs with E-BE, six dogs with E-BM, and five dogs with E- BEBM. Both dogs with E-BE were also diagnosed with CT-BE. Among the six dogs with E-BM, three were diagnosed with CT-BM (50%). For the five dogs with E-BEBM, only two were diagnosed as CT-BEBM (40%). A significant association was found between carina collapse on thoracic CT and the diagnosis of E-BM and E-BEBM (*p* = 0.0015). While all E-BE dogs in this small sample also showed carina collapse, the limited number (n = 2) likely prevented statistical significance for this group. In contrast, no such association was observed between non-tapering airways on thoracic CT and any of the diagnostic groups (*p* = 0.25). Although all E-BE patients exhibited non-tapering airways, this was not consistent in E-BM and E-BEBM groups, and the small sample size further limits interpretability.

## 4. Discussion

The present study reports the clinicopathological and imaging features of 65 dogs with endoscopically diagnosed BE, BM, and BEBM. Across the three groups, clinicopathological differences were minimal. No significant differences in the TCC or neutrophil percentages among the groups was present, but an association was found between the neutrophil count in BALF and the macroscopic inflammatory aspect of the bronchial mucosa. Concomitant bacterial infections were identified in a minority of cases, with no significant differences in their prevalence between the groups. The clinical relevance of differentiating E-BE, E-BM, and E-BEBM remains thus questionable. Furthermore, the diagnostic association between imaging modalities and bronchoscopy was limited.

Most dogs included in this retrospective study were small breed adult or elderly dogs. The mean age of dogs with E-BE, E-BM, and E-BEBM aligns with earlier reports of BE and BM in dogs, although no significant age differences were found between the three groups [1,2,8]). Among the clinical signs evaluated, only the presence of lung crackles on auscultation showed a significant difference between the three groups, being more commonly heard in dogs with bronchial collapse (E-BEBM and E-BM) compared to E-BE. This is not surprising, as lung crackles are well documented in dogs with BM and are attributed to the sudden reopening of collapsed small airways as air rushes in during inspiration [24]. The low number of lung crackles in dogs with E-BE is quite surprising. In humans with BE, lung crackles are frequently auscultated, primarily due to retained secretions in damaged and dilated bronchi [25,26]. Given this well-documented correlation in human patients, a higher occurrence of lung crackles in dogs with E-BE might have been anticipated. Among the 16 dogs diagnosed with E-BE, only 4 showed evidence of mucus accumulation in the bronchi during bronchoscopy, suggesting that secretion retention was not as prominent as typically seen in human cases. Interestingly, of these four dogs, only one also displayed mucus accumulation on CT, indicating lack of concordance between imaging and endoscopic findings. Conversely, another dog exhibited mucus accumulation on CT but not on bronchoscopy, highlighting potential differences in sensitivity between these diagnostic modalities. These findings suggest that either mucus retention is less common in canine E-BE than in human bronchiectasis or that current diagnostic techniques may not be equally effective in detecting secretions in dogs. Interestingly, no significant differences were observed among the three groups in the presence of increased lung sounds, despite such sounds being commonly associated with collapsed airways due to airflow limitation [26,27]. It is worth noting that both human and canine medicine have documented cases of bronchial airflow limitation without the presence of wheezes [26,28]. Finally, as expected, no significant differences were noted between the groups in terms of dyspnea, which is a common feature in both canine BE and BM [2,7].

In this study, 69.2% of dogs showed macroscopic evidence of airway inflammation during bronchoscopy, but no statistically significant differences in the degree of inflammation were observed between the groups. Similarly, at the microscopic level, BALF analysis revealed increased TCC in all dogs with no significant differences in median TCC between groups. The more abundant inflammatory cells in BALF across the three groups were neutrophils. This finding aligns with prior studies, such as the one from [13], who reported a predominance of neutrophilic inflammation in 59 dogs with BM diagnosed by bronchoscopy, and all of them exhibited some degree of macroscopic airway inflammation. Similarly, studies by [1,28] observed that dogs with BE primarily exhibited neutrophilic inflammation, reinforcing the understanding that chronic inflammation is central to the pathogenesis of BE [3]. Despite this, the neutrophil counts did not differ significantly between groups, highlighting that macroscopic and microscopic findings alone cannot distinguish BE, BM, and BEBM. Our study also found that neutrophil percentage in BALF was associated with the degree of macroscopic inflammation observed during bronchoscopy. Dogs with more severe macroscopic inflammation exhibited higher neutrophil percentages, confirming that neutrophils play a key role in the inflammation seen in chronic airway diseases [3,28].

Twenty-one dogs did not show any signs of inflammation on bronchoscopy. Of these, one dog received antibiotics at the time of sample collection, while four dogs had been treated with prednisolone within 2 weeks prior to procedure. The absence of inflammation in these dogs may be attributed to the effects of these medications administered before bronchoscopy. Moreover, even after excluding dogs that had received prednisolone or antibiotics prior to presentation, inflammation and BALF neutrophil percentages remained comparable across groups. It is also worth noting that in older dogs with severely affected airways, the macroscopic appearance of the bronchi may not accurately reflect the degree of inflammation, as the tissue may already be damaged or fibrosed. This could lead to a less pronounced inflammatory appearance compared to younger dogs [12]. However, the mean age of dogs without bronchial inflammation (8.9 years) was similar to the study population’s mean age.

Standard bacterial culture of BALF in dogs with BE and BM has been reported in only a limited number of publications, making the role of bacteria in their development unclear [1,5,24,28]. In this study, 10/65 dogs were diagnosed with concomitant bacterial infection. Human studies have linked species like *Pseudomonas aeruginosa*, *Haemophilus influenzae*, and *Streptococcus pneumoniae* with BE [14,29]. Understanding the specific bacterial species involved is crucial for effective management, as organisms like *P. aeruginosa* have significant prognostic implications in human medicine. These infections are linked to more frequent exacerbations, increased sputum production, higher hospitalization rates, and poorer outcomes in humans [14,30]. In this study, three dogs tested positive for *P. aeruginosa* (two with BE and one with BEBM), with moderate to severe clinical signs. Follow-up showed one dog being euthanized due to respiratory deterioration, while another showed improvement with antibiotics, but it relapsed after discontinuation. Overall, no significant differences in bacterial infection prevalence were found between the groups, nor was there an association between infection and inflammation severity. The use of antibiotics prior to or at the time of enrolment did not affect results. Considering the low frequency of bacterial infections found in this study, it is more probable that these infections are a result of complications or consequences of BE and/or BM, rather than being the primary cause of these conditions.

This study highlights the diagnostic challenges and limitations of thoracic imaging, particularly radiography, in accurately diagnosing BE, BM, and BEBM in dogs. RXs showed poor diagnostic association with endoscopic findings. Additionally, no significant associations were found between radiographic features (e.g., non-tapering airways and carina collapse) and endoscopic diagnoses. The low detection rate of E-BM as R-BM aligns with earlier research, which reports the sensitivity of RX in identifying bronchial collapse to be markedly lower (50–55%) than that of bronchoscopy or CT (70% and 96.6%, respectively) [18,28]. Interestingly, severe cases of BM (classified as BM-III or BEBM-III) consistently exhibited carina collapse on radiographs, suggesting that advanced disease stages may be more readily detectable radiographically. However, the limitations of RX as a primary diagnostic tool for these conditions are increasingly being recognized. Its two-dimensional nature, along with anatomical superimposition, restricts the evaluation of smaller airways, making it less reliable for accurate diagnosis [1,7]. This is particularly evident in dyspneic dogs, where obtaining both inspiratory and expiratory radiographs for comparison is often not feasible, further reducing their diagnostic value. Thoracic CT images showed varying levels of diagnostic association with endoscopy-based diagnoses. Interestingly, CT images demonstrated a significant association with endoscopy in detecting main bronchi collapse, suggesting it may be a more reliable tool for detecting such specific structural bronchial changes compared to radiography. However, no significant association was found between the presence of non-tapering airways on CT and the endoscopic diagnoses. Due to the limited number of dogs undergoing CT in this study, these findings should be interpreted with caution. The comparison between CT and bronchoscopy in diagnosing BE, BM, and BEBM warrants further investigation. Notably, spare information exists in the veterinary literature regarding imaging criteria (either radiographic or CT-based) for diagnosing BM. In this retrospective study, the presence or absence of BM on CT for example was based solely on the aspect of the main bronchi, excluding the smaller bronchi, which were not assessed by CT. This contrasts with endoscopic criteria, where both the main and smaller bronchi are considered. Therefore, so far, CT and bronchoscopy should be considered as examinations that complement each other.

The BAR is considered a key criterion for diagnosing BE on CT, with a threshold >2.0 during the expiratory phase suggested by Cannon et al., 2009 [11]. In our study, focusing on the expiratory phase alone, a BAR >2.0 was observed in only 30% of measurements in dogs with BE. This contrasts with the findings of [11], although a later study [19] reported that a BAR <2.0 could occasionally be observed during the expiratory phase in dogs with BE. Despite the limited number of dogs that underwent CT in this study, it remains important to consider the classical criteria, such as the presence of non-tapering airways and bronchial wall thickening, in order to make a diagnosis of BE (or BEBM), since a BAR of <2.0 does not necessarily exclude a diagnosis of BE, particularly when other imaging and clinical findings support the diagnosis. In the BEBM group, the median BAR did not exceed 1.5 in any lobe. This may be attributed to the presence of bronchomalacia in this group, which causes greater bronchial collapse during expiration compared to dogs with BE alone. Such collapse may reduce the apparent bronchial diameter, thus lowering the BAR values in dogs with BEBM. However, no significant associations were found between BAR measurements and CT diagnoses of BE or BEBM in this study. When combining the CT-BE and CT-BEBM groups, significant differences in the mean BAR values between inspiratory and expiratory phases were noted in the cranial lung lobes and the right middle lung lobe, suggesting that dynamic airway changes can be detected in these regions. However, no significant differences in BAR measurements across lung lobes were detected within the CT-BE or CT-BEBM groups independently. These findings align with [11,19], though it is important to note that those studies focused solely on dogs without clinical pulmonary disease and those with BE. Overall, these results suggest that respiratory phase variations may influence BAR measurements and highlight the importance of obtaining paired inspiratory and expiratory CT. The dynamic nature of airway collapse may not be fully appreciated with static imaging alone [7]. Despite its clinical utility, the BAR has several limitations that may have influenced measurements in this study. Slice thickness artifacts could have contributed to measurement errors. Additionally, oblique orientation of bronchi and vessels may have affected the accuracy of BAR calculations. The assumption of a normal pulmonary artery in BAR calculations may not always hold true. In diseased lung lobes, pulmonary arterial blood flow may be decreased, with compensatory increased flow to unaffected lobes due to altered regional ventilation. This redistribution of blood flow could result in a higher BAR in diseased lobes and a lower BAR in unaffected lobes, potentially skewing the interpretation of results. Moreover, four dogs in which the BAR was measured showed signs of mild to moderate pulmonary hypertension (PH). The presence of PH may have significantly influenced BAR measurement, as increased pulmonary arterial pressure leads to pulmonary artery dilation. This arterial enlargement can artificially lower the BAR, even if the bronchial diameter remains unchanged. Consequently, dogs with true BE may present with a falsely normal or low BAR, potentially resulting in the underdiagnosis of BE when relying on BAR criteria alone. Another methodological limitation is the inability to calculate BAR at the exact locations specified by [11]), particularly in the caudal lung lobes. In many dogs, we were unable to measure the BAR at the level of the 11th rib in cross-sectional images because there was no lung parenchyma left at this point, or the lung stopping cranially on some animals due to their small size and/or obesity. In cases where parenchyma was present, the bronchi were often too small (e.g., ~1 mm in diameter), approaching the spatial resolution limits of most clinical CT scanners, which hampered reliable detection of subtle differences between inspiratory and expiratory phases. Consequently, we initially attempted to perform BAR measurements at more cranial levels in these lobes. However, this may have affected measurement accuracy, as caudal lung regions might exhibit less dynamic airway variation due to differences in compliance, airway morphology, and gravitational influence. The cranial shift in measurement location also meant that smaller bronchi were assessed, which may inherently show less collapsibility and thus reduce the likelihood of detecting significant differences. Given these combined challenges, we ultimately chose not to include BAR measurements from the caudal lung lobes.

This study contains some limitations. First, during the time period of the retrospective study, bronchoscopies were realized by various specialists without standardized recording protocol. Parts of the lower airways may not have been recorded leading to misclassification of the patients. However, this limitation is of minor importance in our opinion. Recordings might not be standardized, and thus, the complete respiratory tract might not be visualized, but it is a general accepted rule under the different clinicians that all macroscopic abnormalities are recorded and can thus be evaluated retrospectively. Secondly, the grading system, only based on endoscopy videos, remains a subjective evaluation, even though all videos were evaluated independently by two diplomates experienced in respiratory medicine. In 16 out of 65 dogs, interobserver disagreement occurred, necessitating a re-evaluation by both observers together. This was followed by a consensual appraisal conducted blindly to the previous assessments. Next, due to the retrospective nature of this study, no standardized validated questionnaire was used to evaluate the degree and severity of the clinical symptoms, which remained thus subjective. Additionally, due to the retrospective nature of this study, dogs receiving anti-inflammatory drugs and antimicrobials were included. The only dog receiving antimicrobial treatment was excluded from the statistical analysis related to microbiological and inflammation data. However, the 13 dogs that had been on anti-inflammatory drugs within the two weeks prior to presentation were not excluded, and their results regarding inflammation and clinical signs may have been influenced by the recent drug administration. Moreover, the depth of anesthesia was not standardized, and in some cases, it was more superficial, leading to occasional coughing during video recording. While we made efforts to avoid capturing videos during coughing, this remains a minor limitation that could potentially affect image quality and interpretation, as we were focusing on dynamic images. Lastly, CT allows for quantitative measurement such as the BAR. However, a limitation of a quantitative measure such as the BAR is interobserver variability. In the majority of the cases, the margins of the bronchus and the airways were well delineated, so the chance of measurement error was less likely. In order to confirm objectively bronchial collapsibility during paired CT, the bronchial collapsibility can be calculated [31]. This feature was not included during re-evaluation of the CT images in this study.

## 5. Conclusions

This study describes an association between BE and BM based on endoscopy findings. However, it highlights limited clinicopathological distinctions between endoscopy-based diagnoses of BE, BM, and BEBM, as well as the absence of significant differences in BALF analysis and bacteriological findings across the three groups. The limited diagnostic agreement between imaging modalities and bronchoscopy further highlights the challenges in differentiating these conditions based solely on clinical, endoscopic, and imaging data. These findings raise questions about the clinical relevance of distinguishing between these endoscopic diagnoses, as they currently do not provide clear guidance for selecting specific treatment strategies. Treatment decisions should thus rely on a thorough assessment of all available data, including BALF cytological analysis, bacterial culture results on BALF, and qPCR results on BALF, rather than strictly relying on the endoscopic diagnosis. Importantly, these results do not support the routine use of antibiotics for all animals with these diagnoses.

Prospective studies are needed to determine whether therapeutic approaches and follow-up care should vary between these entities and to evaluate the impact of tailored treatments on clinical outcomes. While the macroscopic endoscopic distinctions between the three groups may not appear clinically relevant at this stage, future investigations may reveal a role for these differences in guiding treatment strategies.

## Figures and Tables

**Table 1:** Signalment, imaging findings, and final diagnosis for each dog.

Endoscopy Diagnosis	Dog	Age (year)	Sex	Breed	RX Findings	CT Findings	RX Diagnosis/CT Diagnosis	Final Etiology
**E-BE**	1	7	FN	Beagle	NTA+, S+ C+, CMB−, PC−, BWTh+, BWO−	N/A	R-BE	PB
	2	5	FN	Bouvier des Flandres	NTA+, S+ C+, CMB+, PC+, BWTh+, BWO+	N/A	R-BEBM	CB
	3	12	MN	Crossbreed	N/A	N/A	/	EBP
	4	13	MN	Crossbreed	NTA+, S− C+, CMB+, PC−, BWTh+, BWO−	N/A	R-BEBM	CB
	5	11	MN	Crossbreed	N/A	N/A	/	CB
	6 ^$^	10	FN	Crossbreed	NTA+, S+ C−, CMB+, PC+, BWTh+, BWO−	N/A	R-BEBM	CB
	7	12	FN	Dachshund	NTA−, S− C−, CMB+, PC−, BWTh+, BWO+	N/A	R-BM	CB
	8	6	FN	Dachshund	N/A	N/A	/	CB
	9	5	ME	Fox Terrier	N/A	N/A	/	CB
	10	15	FN	Jack Russel Terrier	NTA+, S− C+, CMB−, PC−, BWTh+, BWO−	N/A	R-BE	EBP
	11	10	MN	Malinois	NTA+, S+ C+, CMB+, PC+, BWTh+, BWO−	NTA+, S+ C−, CMB−, PC+, BWTh+, BWO+, GGO+	R-BEBM/CT-BE	CB
	12	9	FN	Maltese	NTA−, S− C−, CMB−, PC−, BWTh+, BWO−	N/A	-/-	CB
	13	8	ME	West Highland White Terrier	N/A	NTA+, S− C+, CMB−, PC−, BWTh+, BWO+, GGO+	CT-BE	CB
	14 ^$^	3	ME	Whippet	NTA−, S− C−, CMB−, PC−, BWTh+, BWO−	N/A	-/-	EBP
	15	4	MN	Whippet	NTA−, S− C−, CMB−, PC−, BWTh+, BWO−	N/A	-/-	EBP
	16	9	FN	Yorkshire Terrier	NTA+, S− C+, CMB+, PC−, BWTh−, BWO−	N/A	R-BEBM	CB
**E-BM 25–50%**	17	8	FN	Cavalier King Charles Spaniel	NTA−, S− C−, CMB−, PC−, BWTh−, BWO−	N/A	-/-	CB
	18	12	FE	Crossbreed	NTA−, S− C−, CMB−, PC−, BWTh+, BWO−	N/A	-/-	CB
	19	12	ME	Crossbreed	NTA+, S− C+, CMB−, PC+, BWTh+, BWO+	N/A	R-BE	CB
	20	13	MN	Crossbreed	NTA+, S+ C+, CMB−, PC−, BWTh+, BWO−	N/A	R-BE	CB
	21 *	11	FN	Griffon Bruxellois	NTA+, S+ C+, CMB+, PC+, BWTh+, BWO−	NTA+, S+ C−, CMB+, PC−, BWTh+, BWO+, GGO+	R-BEBM/CT-BEBM	CB
	22	4	FN	Pomerian	NTA−, S− C−, CMB−, PC−, BWTh+, BWO−	N/A	-/-	CB
	23	9	FN	Sheltie	NTA+, S− C+, CMB+,PC−, BWTh+, BWO−	N/A	R-BEBM	CB
	24	7	MN	Shih Tzu	NTA+, S− C+, CMB−, PC−, BWTh+, BWO−	NTA−, S− C−, CMB+, PC−, BWTh−, BWO−, GGO−	R-BE/CT-BM	CB
	25	7	FN	Whippet	NTA+, S− C+, CMB+, PC−, BWTh+, BWO−	N/A	R-BEBM	CB
	26	7	MN	Yorkshire Terrier	NTA−, S− C−, CMB−, PC−, BWTh+, BWO−	N/A	-/-	CB
**E-BM 50–75%**	27	8	FN	Crossbreed	NTA−, S− C−, CMB−, PC−, BWTh+, BWO−	N/A	-/-	CB
	28	13	MN	Labrador Retriever	N/A	N/A	-/-	CB
	29	13	MN	Maltese	N/A	N/A	-/-	CB
	30	11	MN	Poodle	NTA−, S− C−, CMB−, PC−, BWTh+, BWO+	N/A	-/-	CB
	31	12	ME	Poodle	NTA+, S+ C+, CMB−, PC−, BWTh+, BWO−	N/A	R-BE	CB
	32 ^$^	8	FN	Sheltie	NTA+, S+ C−, CMB−, PC+, BWTh+, BWO−	NTA−, S− C−, CMB+, PC+, BWTh+, BWO−, GGO+	R-BE/CT-BM	CB
	33	8	FN	West Highland White Terrier	N/A	N/A	/	IPF
	34 ^$^	13	FN	Yorkshire Terrier	N/A	N/A	/	CB
	35 ^$^	7	MN	Yorkshire Terrier	N/A	N/A	/	CB
	36	11	MN	Yorkshire Terrier	NTA−, S− C−, CMB+, PC−, BWTh+, BWO−	N/A	R-BM	CB
	37	13	ME	Yorkshire Terrier	N/A	N/A	/	CB
**E-BM > 75%**	38	2	FN	Cavalier King Charles Spaniel)	NTA−, S− C−, CMB+, PC+, BWTh−, BWO−	NTA−, S− C−, CMB+, PC+, BWTh+, BWO−, GGO+	R-BM/CT-BM	CB
	39 ^$^	8	FN	Crossbreed	N/A	NTA+, S+ C−, CMB+, PC−, BWTh+, BWO−, GGO+	CT-BEBM	CB
	40 ^$^	10	FN	Jack Russel Terrier	N/A	N/A	/	CB
	41 ^$^	10	FN	Jack Russel Terrier	NTA+, S− C+, CMB+, PC+, BWTh+, BWO−	NTA−, S− C−, CMB+, PC−, BWTh+, BWO−, GGO+	R-BEBM/CT-BM	CB
	42	12	FN	Maltese	N/A	N/A	/	CB
	43	17	ME	Maltese	N/A	N/A	/	CB
	44 ^$^	7	ME	Pomerian	NTA+, S+ C−, CMB+, PC+, BWTh+, BWO−	N/A	R-BEBM	CB
	45 ^$^	9	MN	Shih Tzu	NTA+, S+ C−, CMB+, PC−, BWTh+, BWO−	N/A	R-BEBM	CB
	46	11	FN	Yorkshire Terrier	N/A	N/A	/	CB
	47	14	MN	Yorkshire Terrier	N/A	N/A	/	CB
**E-BEBM 25–50%**	48	7	ME	Leonberger	NTA+, S+ C+, CMB−, PC+, BWTh+, BWO−	N/A	R-BE	CB
	49	9	FE	Münsterländer	NTA+, S+ C+, CMB−, PC−, BWTh+, BWO−	N/A	R-BE	CB
	50	8	FN	Samoyed	NTA+, S− C+, CMB+, PC+, BWTh+, BWO−	N/A	R-BEBM	EBP
	51	11	ME	West Highland White Terrier	N/A	NTA−, S− C−, CMB+, PC+, BWTh+, BWO−, GGO+	CT-BM	IPF
	52	7	ME	Whippet	N/A	N/A	/	CB
**E-BEBM 50–75%**	53 ^$^	11	FN	Basset Fauve de Bretagne	NTA-, S− C−, CMB+, PC+, BWTh+, BWO−	N/A	R-BM	CB
	54	13	ME	Beagle	NTA+, S− C+, CMB−, PC−, BWTh+, BWO−	N/A	R-BE	CB
	55	8	MN	Chihuahua	NTA−, S− C−, CMB−, PC−, BWTh−, BWO−	N/A	-/-	CB
	56	13	MN	Crossbreed	NTA+, S+ C−, CMB+, PC+, BWTh+, BWO−	N/A	R-BEBM	CB
	57	6	FN	Crossbreed	NTA+, S+ C+, CMB−, PC+, BWTh+, BWO+	N/A	R-BEBM	CB
	58	9	ME	Sheltie	N/A	NTA−, S− C−, CMB+, PC−, BWTh+, BWO−, GGO+	CT-BM	CB
	59 ^$^	13	MN	Shih Tzu	NTA+, S− C+, CMB+, PC+, BWTh+, BWO+	N/A	R-BEBM	CB
	60	10	FN	Shih Tzu	NTA−, S− C−, CMB+, PC−, BWTh+, BWO−	N/A	R-BM	CB
	61	14	MN	Tibetan Terrier	NTA+, S+ C−, CMB−, PC−, BWTh+, BWO−	NTA+, S+ C+, CMB+, PC+, BWTh+, BWO−, GGO+	R-BE/CT-BEBM	CB
	62	13	FN	West Highland White Terrier	N/A	NTA−, S− C−, CMB+, PC−, BWTh+, BWO−, GGO+	CT-BM	IPF
	63	10	FN	West Highland White Terrier	N/A	NTA+, S+ C−, CMB+, PC+, BWTh+, BWO−, GGO+	CT-BEBM	IPF
**E-BEBM > 75%**	64	10	ME	Shih Tzu	NTA−, S− C−, CMB+, PC−, BWTh−, BWO−	N/A	R-BM	CB
	65 ^$^	8	MN	Yorkshire Terrier	NTA+, S− C+, CMB+, PC−, BWTh+, BWO+	N/A	R-BEBM	CB

Abbreviations: BALF = bronchoalveolar lavage fluid; FN = female neutered; MN = male neutered; ME = male intact; FE = female intact; E-BE = endoscopic bronchiectasis, E-BM = endoscopic bronchomalacia; E-BEBM = endoscopic bronchiectasis + bronchomalacia; + = present; − = absent; * indicates that the dog received antimicrobial treatment at the moment of presentation; ^$^ indicates that the dog received corticosteroids at anti-inflammatory dosage within 2 weeks prior to presentation; RX = radiography; CT = computed tomography; NTA = non-tapering airways; S = saccular bronchiectasis; C = cylindrical bronchiectasis; CMB = collapse of the main bronchi; PC = pulmonary consolidation; BWTh = bronchial wall thickening; BWO = bronchial wall occlusion; GGO = ground-glass opacification; R-BE = radiographic diagnosis of bronchiectasis; R-BM = radiographic diagnosis of bronchomalacia; R-BEBM = radiographic diagnosis of bronchiectasis + bronchomalacia; CT-BE = computed tomography diagnosis of bronchiectasis; CT-BM = computed tomography diagnosis of bronchomalacia; CT-BEBM = computed tomography diagnosis of bronchiectasis + bronchomalacia; PB = parasitic bronchitis; CB = chronic bronchitis; EBP = eosinophilic bronchopneumopathy; IPF = idiopathic pulmonary fibrosis.

**Table 2:** Clinical signs of the dogs.

Clinical Sign	E-BE (*n* = 16) n (%)	E-BM (*n* = 31) n (%)	E-BEBM (*n* = 18) n (%)	*p*-Value
Cough				0.57
Mild to moderate	9 (13.85%)	16 (24.62%)	7 (10.77%)	
Severe	7 (10.77%)	15 (23.08%)	11 (16.92%)	
Exercise intolerance	4 (25%)	16 (51.6%)	11 (61.1%)	0.091
Dyspnea	2 (12.5%)	10 (32.3%)	8 (44.4%)	0.13
Increased lung sounds	/	7 (22.6%)	2 (11.1%)	0.097
Lung crackles	1 (6.3%)	8 (25.8%)	9 (50%)	0.017 *

The results are presented as number (%) and statistical differences are indicated by an asterisk (*). Abbreviations: E-BE = endoscopy-based diagnosis of bronchiectasis, E-BM = endoscopy-based diagnosis of BM; E-BEBM = endoscopy-based diagnosis of bronchiectasis/bronchomalacia.

**Table 3:** Distribution of bacteria retrieved from cultures or PCR of bronchoalveolar lavage fluid in dogs with bronchiectasis, bronchomalacia, and bronchiectasis/bronchomalacia.

Bacteria	E-BE (n = 16)	E-BM (n = 31)	E-BEBM (n = 18)	Total (n = 65)
*Escherichia* sp.	2 (dogs 7 & 9)	0	0	2
*Pseudomonas* sp.	2 (dogs 2 & 16)	0	1 (dog 60)	3
*Serratia* sp.	0	2 (dogs 19 and 26)	0	2
*Streptococcus* sp.	0	0	1 (dog 48)	1
*Bordetella bronchiseptica (qPCR)*	0	0	1 (dog 64)	1
*Mycoplasma cynos (qPCR)*	1 (dog 14)	0	0	1
**Total number of dogs**	**5**	**2**	**3**	**10**

Abbreviations: E-BE = endoscopy-based diagnosis of bronchiectasis, E-BM = endoscopy-based diagnosis of BM; E-BEBM = endoscopy-based diagnosis of bronchiectasis/bronchomalacia; qPCR = quantitative polymerase chain reaction; sp. = species.

**Table 4:** Description of the bronchial-to-arterial ratio of cross-sectional CT images of the inspiration and expiration phases in dogs diagnosed with BE or BEBM based on CT. The results are presented as median with the interquartile range [Q1; Q3].

Location	Median BAR [Interquartile Range Q1; Q3] (Inspiratory)	Median BAR [Interquartile Range Q1; Q3] (Expiratory)
**R cranial lobe 4th rib**		
CT-BE	2.4 [2.3; 2.6]	2.2 [1.9; 2.5]
CT-BEBM	2.1 [1.8; 2.3]	1.5 [1.0; 1.6]
*p*-value	0.56	0.081
**L cranial lobe 4th rib**		
CT-BE	2.4 [2.3; 2.5]	2.0 [1.8; 2.2]
CT-BEBM	1.6 [1.1; 1.65]	0.8 [0.7; 0.8]
*p*-value	0.081	0.081
**R middle lobe**		
CT-BE	2.2 [1.4; 2.9]	1.9 [1.4; 2.3]
CT-BEBM	1.6 [1.4; 2.5]	1.3 [1.2; 1.5]
*p*-value	0.85	0.33
**L cranial (caudal part)**		
CT-BE	1.2 [1.2; 1.3]	1.5 [1.4; 1.7]
CT-BEBM	1.0 [1.0; 1.0]	0.8 [0.8; 1.1]
*p*-value	0.18	0.081
**R accessory lobe**		
CT-BE	1.5 [1.3; 1.7]	1.4 [1.3; 1.5]
CT-BEBM	1.4 [0.9; 1.5]	1.2 [1.1; 1.2]
*p*-value	0.85	0.081

Abbreviations: BAR = bronchial-to-arterial ratio; SD = standard deviation; R = right; L = left; CT-BE = computed tomography-based diagnosis of bronchiectasis; CT-BEBM = computed tomography-based diagnosis of bronchiectasis/bronchomalacia.

## Data Availability

Data is contained within the article.

## Abbreviations

The following abbreviations are used in this manuscript:

BALF	Bronchoalveolar fluid analysis
BAR	Bronchial to arterial ratio
BE	Bronchiectasis
BEBM	Bronchiectasis and bronchomalacia
BM	Bronchomalacia
CFU	Colony-forming units
CRP	C-reactive protein
CT	Computed tomography
CT-BE	Computed-tomography-based diagnosis of bronchiectasis
CT-BEBM	Computed-tomography-based diagnosis of bronchiectasis and bronchomalacia
CT-BM	Computed-tomography-based diagnosis of bronchomalacia
DCC	Differentiated cellular count
E-BE	Endoscopy-based diagnosis of bronchiectasis
E-BEBM	Endoscopy-based diagnosis of bronchiectasis and bronchomalacia
E-BM	Endoscopy-based diagnosis of bronchomalacia
PH	Pulmonary hypertension
qPCR	Quantitative polymerase chain reaction
R-BE	Radiography-based diagnosis of bronchiectasis
R-BEBM	Radiography-based diagnosis of bronchiectasis and bronchomalacia
R-BM	Radiography-based diagnosis of bronchomalacia
RX	Thoracic radiography
SD	Standard deviation
TCC	Total cellular count
